# Analysis and Experimental Kinematics of a Skid-Steering Wheeled Robot Based on a Laser Scanner Sensor

**DOI:** 10.3390/s150509681

**Published:** 2015-04-24

**Authors:** Tianmiao Wang, Yao Wu, Jianhong Liang, Chenhao Han, Jiao Chen, Qiteng Zhao

**Affiliations:** Robotics Institute, Beihang University, Beijing 100191, China; E-Mails: wtm_itm@263.net (T.W.); dommy_leung@263.net (J.L.); shasmyan@sina.cn (C.H.); chenjiao_1989@yeah.net (J.C.); zqt_buaa@126.com (Q.Z.)

**Keywords:** mobile robot, laser scanner, skid-steering, instantaneous centers of rotation, experimental kinematics, dynamic model

## Abstract

Skid-steering mobile robots are widely used because of their simple mechanism and robustness. However, due to the complex wheel-ground interactions and the kinematic constraints, it is a challenge to understand the kinematics and dynamics of such a robotic platform. In this paper, we develop an analysis and experimental kinematic scheme for a skid-steering wheeled vehicle based-on a laser scanner sensor. The kinematics model is established based on the boundedness of the instantaneous centers of rotation (ICR) of treads on the 2D motion plane. The kinematic parameters (the ICR coefficient χ, the path curvature variable λ and robot speed v), including the effect of vehicle dynamics, are introduced to describe the kinematics model. Then, an exact but costly dynamic model is used and the simulation of this model’s stationary response for the vehicle shows a qualitative relationship for the specified parameters χ and λ. Moreover, the parameters of the kinematic model are determined based-on a laser scanner localization experimental analysis method with a skid-steering robotic platform, Pioneer P3-AT. The relationship between the ICR coefficient χ and two physical factors is studied, *i.e.*, the radius of the path curvature λ and the robot speed v. An empirical function-based relationship between the ICR coefficient of the robot and the path parameters is derived. To validate the obtained results, it is empirically demonstrated that the proposed kinematics model significantly improves the dead-reckoning performance of this skid–steering robot.

## 1. Introduction

Skid-steering motion is widely used for wheeled and tracked mobile robots [1]. Steering in this way is based on controlling the relative velocities of the left and right side drives. The robot turning requires slippage of the wheels for wheeled vehicles. Due to their identical steering mechanisms, wheeled and tracked skid-steering vehicles share many properties [2,3].

Like differential steering, skid steering leads to high maneuverability [4,5], and has a simple and robust mechanical structure, leaving more room in the vehicle for the mission equipment [3,6]. In addition, it has good mobility on a variety of terrains, which makes it suitable for all-terrain missions.

However, this locomotion scheme makes it difficult to develop kinematic and dynamic models that can accurately describe the motion. It is very difficult for the skid-steering kinematics to predict the exact motion of the vehicle only from its control inputs. As a result, the kinematics models with pure rolling and no-slip assumptions for non-holonomic wheeled vehicles cannot apply in this case [2]. Furthermore, other disadvantages are that the motion tends to be energy inefficient, difficult to control, and for wheeled vehicles, the tires tend to wear out faster [6,7]. 

Some previous studies have discussed the dynamic control of skid-steering mobile robots. A dynamic model was presented for a skid-steering four-wheel robot and a non-holonomic constraint between the robot’s lateral velocity and yaw rate was considered in [5]. A perfect wheel-ground interaction was assumed. A simple Coulomb friction model was used to capture the wheel-ground interaction and a nonlinear feedback controller was designed to track the desired path [6]. Yu and Ylaya Chuy developed a skid-steering mobile robot dynamic model for general 2D motion and linear 3D motion [8,9]. This model was based on the functional relationship of shear stress to shear displacement, which is different from the previous Coulomb-friction-based model [5,6]. It needs a lot of computational effort to calculate a complex dynamics model in real-time, for example, this work requires a number of integral operations, so the dynamic models for skid-steering may result too costly for real-time motion control and dead-reckoning.

In the meantime, Maalouf *et al*. [10] and Kozlowski [11] separately considered the kinematics for the relation between drive velocities and vehicle velocities without concerning themselves with major skid effects. As we know, wheel slip plays a critical role in the kinematic and dynamic modeling of skid-steering mobile robots. The slip information provides a connection between the wheel rotation velocity and the linear motion of the robot platform. With an extended Kalman filter, the slip estimation was performed from actual inertial readings and a kinematics model of the vehicle relates the slip parameters to the track velocities [12]. Furthermore, an experimental method was developed to determine the slip ratios. The slip coefficients of tracks were modeled as an exponential function of path radius [13].

An extra trailer was designed to study the kinematic relationship for simultaneous localization and mapping (SLAM) applications. It was concluded that an ideal differential-driven kinematics model for wheeled robot cannot be used for skid-steering robots [14,15]. Meanwhile, geometric analogy with an ideal differential-driven wheeled mobile robot was studied [2,7]. Experimental validations have been conducted for both tracked vehicles and skid-steering mobile robots. These correspond to the position of ideal differential drive wheels for a particular terrain. This is based on the fact that tread ICR values are dynamics-dependent, but they lie within a bounded area at moderate speeds. A group of constant kinematic parameters were derived as optimized values for the tread ICR on the plane. Specifically, we find that tread ICR values vary with the speed of the robot and the path curvature, so it is necessary to further describe the relationship between tread ICR values and curvature of the path and the vehicle speed.

Building upon the research by Mandow [2] and Moosavian [13], we develop an experimental kinematics model for a skid-steering mobile robot. The kinematics model based on ICR of both treads on the motion plane is used [2], and we consider that tread ICR values change with the speed of the robot and the path curvature by explicitly considering slip ratio [13]. A dynamic model based on the research by Yu [8,9] and Wong [16,17] is developed for a simulation in order to estimate a potential kinematics relationship. Because a laser scanner is accurate and efficient for mobile robot localization and dead-reckoning [18,19], with a laser-scanner-based experimental method, an approximating function is derived to describe the relationship between the ICR values of the robot and the radius of curvature of the path and speed of the robot. 

The main contribution in this paper is that the new analysis and experimental kinematic scheme of the skid-steering robot reveal the underlying kinematic relationship between the ICR coefficient of the robot and the path parameters. The simulation based on a dynamic model analysis shows a qualitative relationship among the parameters theoretically specified before the experiment. An empirical function relationship between the ICR values of the robot and the path parameters is derived with this laser-scanner-based experimental method. Dead-reckoning performance shows that the empirical function kinematics model improves the motion estimation accuracy significantly. This laser-scanner-based method is easy to operate and does not add extra sensors or change the vehicle mechanical structure and control system. The proposed model and analysis approach can be further used for robot control, as an exact kinematics control can be used for a skid-steering robot [13].

The remainder of this paper is organized as follows: Section 2 presents the kinematic and dynamic modeling of a four-wheel skid-steering mobile robot. A dynamic model based simulation to explain the potential kinematics relationship is proposed. In Section 3 the laser-scanner-based localization method is presented and the experiment results and analyses are given.

## 2. Model Analysis and Simulation

### 2.1. Kinematical Analogy of Skid-Steering with Differential Drive 

**Figure 1:**
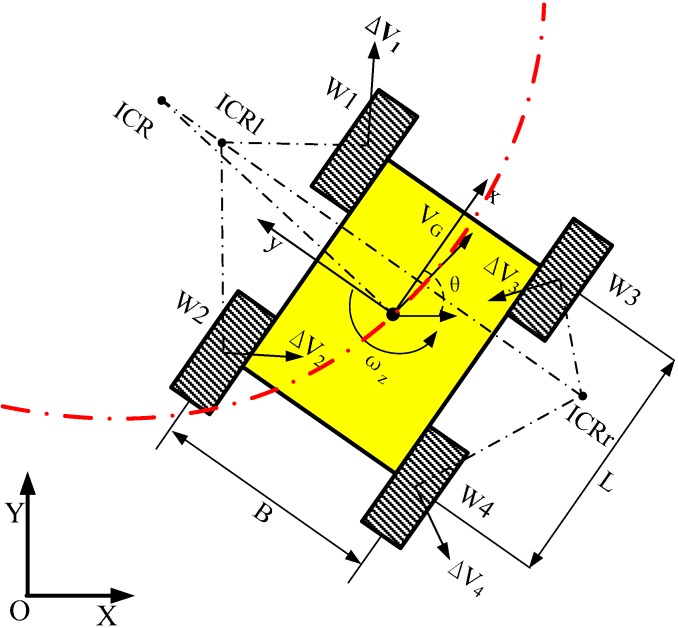
The kinematics schematic of skid-steering mobile robot.

Figure 1 shows the kinematics schematic of a skid-steering robot. We consider the following model assumptions:

(1) the mass center of the robot is located at the geometric center of the body frame;
(2) the two wheels of each side rotate at the same speed;
(3) the robot is running on a firm ground surface, and four wheels are always in contact with the ground surface.

We define an inertial frame (X,Y) (global frame) and a local (robot body) frame (x,y), as shown in Figure 1. Suppose that the robot moves on a plane with a linear velocity expressed in the local frame as $v={\left(v_{x},v_{y},0\right)}^{T}$ and rotates with an angular velocity vector $\omega ={\left(0,0,\omega_{z}\right)}^{T}$. If $q={\left(X,Y,\theta \right)}^{T}$ is the state vector describing generalized coordinate of the robot (*i.e.*, the COM position, X and Y, and the orientation θ of the local coordinate frame with respect to the inertial frame), then $\dot{q}={\left(\dot{X},\dot{Y},\dot{\theta }\right)}^{T}$ denotes the vector of generalized velocities. It is straightforward to calculate the relationship of the robot velocities in both frames as follows [6]:

(1) $$\left[\begin{array}{c} \dot{X}\\ \dot{Y}\\ \dot{\theta } \end{array}\right]=\left[\begin{array}{ccc} cos\theta & -sin\theta & 0\\ sin\theta & cos\theta & 0\\ 0 & 0 & 1 \end{array}\right]\left[\begin{array}{c} v_{x}\\ v_{y}\\ \omega_{z} \end{array}\right]$$

Let $\omega_{i}$, *i* = 1,2,3,4 denote the wheel angular velocities for front-left, rear-left, front-right and rear-right wheels, respectively. From assumption (2), we have:

(2) $$\begin{array}{c} \omega_{L}=\omega_{1}=\omega_{2},\;\;\omega_{R}=\omega_{3}=\omega_{4} \end{array}$$

Then the direct kinematics on the plane can be stated as follows:

(3) $$\left[\begin{array}{c} v_{x}\\ v_{y}\\ \omega_{z} \end{array}\right]=f\left[\begin{array}{c} \omega_{l}r\\ \omega_{r}r \end{array}\right]$$

where $v=\left(v_{x},v_{y}\right)$ is the vehicle’s translational velocity with respect to its local frame, and $\omega_{z}$ is its angular velocity, r is the radius of the wheel.

When the mobile robot moves, we denote instantaneous centers of rotation (ICR) of the left-side tread, right-side tread, and the robot body as $ICR_{l}$, $ICR_{r}$ and $ICR_{G}$, respectively. It is known that $ICR_{l}$, $ICR_{r}$ and $ICR_{G}$ lie on a line parallel to the *x*-axis [7,16]. We define the *x-y* coordinates for $ICR_{l}$, $ICR_{r}$ and $ICR_{G}$ as $\left(x_{l},y_{l}\right)$, $\;\left(x_{r},y_{r}\right)$, and $\left(x_{G},y_{G}\right)$, respectively.

Note that treads have the same angular velocity $\omega_{z}$ as the robot body. We can get the geometrical relation:

(4) $$y_{G}=\frac{v_{x}}{\omega_{z}}$$

(5) $$y_{l}=\frac{v_{x}-\omega_{l}r}{\omega_{z}}$$

(6) $$y_{r}=\frac{v_{x}-\omega_{r}r}{\omega_{z}}$$

(7) $$x_{G}=x_{l}=x_{r}=-\frac{v_{y}}{\omega_{z}}$$

From Equations (4)–(7), the kinematics relation (3) can be represented as:

(8) $$\left[\begin{array}{c} v_{x}\\ v_{y}\\ \omega_{z} \end{array}\right]=J_{\omega }\left[\begin{array}{c} \omega_{l}r\\ \omega_{r}r \end{array}\right]$$

Where the elements of matrix ${\text{J}}_{\omega }$ depend on the tread ICR coordinates:

(9) $$J_{\omega }=\frac{1}{y_{l}-y_{r}}\left[\begin{array}{cc} -y_{r} & y_{l}\\ x_{G} & -x_{G}\\ -1 & 1 \end{array}\right]$$

If the mobile robot is symmetrical, we can get a symmetrical kinematics model (*i.e.*, the ICRs lie symmetrically on the *x*-axis and $x_{G}\;$ = 0), so matrix $J_{\omega }$ can be written as the following form:

(10) $$J_{\omega }=\frac{1}{2y_{0}}\left[\begin{array}{cc} y_{0} & y_{0}\\ 0 & 0\\ -1 & 1 \end{array}\right]$$

where $y_{0}=y_{l}=-y_{r}$ is the instantaneous tread ICR value. Noted that $v_{l}=\omega_{l}r,\;\;\;v_{r}=\omega_{r}r$, for the symmetrical model, the following equations can be obtained:

(11) $$\{\begin{array}{l} v_{x}=\frac{\omega_{l}r+\omega_{r}r}{2}=\frac{v_{l}+v_{r}}{2}\\ v_{y}=0\\ \omega_{z}=\frac{-\omega_{l}r+\omega_{r}r}{2y_{0}}=\frac{-v_{l}+v_{r}}{2y_{0}} \end{array}$$

Noted $v_{y}=0$, so that $v_{G}=v_{x}$. We can get the instantaneous radius of the path curvature:

(12) $$R=\frac{v_{G}}{\omega_{z}}=\frac{v_{G}}{\omega_{z}}=\frac{v_{l}+v_{r}}{-v_{l}+v_{r}}y_{0}$$

A non-dimensional path curvature variable λ is introduced as the ratio of sum and difference of left- and right-side’s wheel linear velocities [1], namely:

(13) $$\lambda =\frac{v_{l}+v_{r}}{-v_{l}+v_{r}}\;$$

and we can rewrite Equation (12) as:

(14) $$R=\frac{v_{l}+v_{r}}{-v_{l}+v_{r}}y_{0}=\lambda y_{0}$$

We use a similar index as in Mandow’s work [2,7], then an ICR coefficient χ can be defined as:

(15) $$\chi \;=\frac{y_{l}-y_{r}}{B}=\frac{2y_{0}}{B},\;\;\chi \geq 1$$

where B denotes the lateral wheel bases, as illustrated in Figure 1. The ICR coefficient χ is equal to 1 when no slippage occurs (ideal differential drive). Note that the locomotion system introduces a non-holonomic restriction in the motion plane because the non-square matrix $J_{\omega }$ has no inverse. 

**Figure 2:**
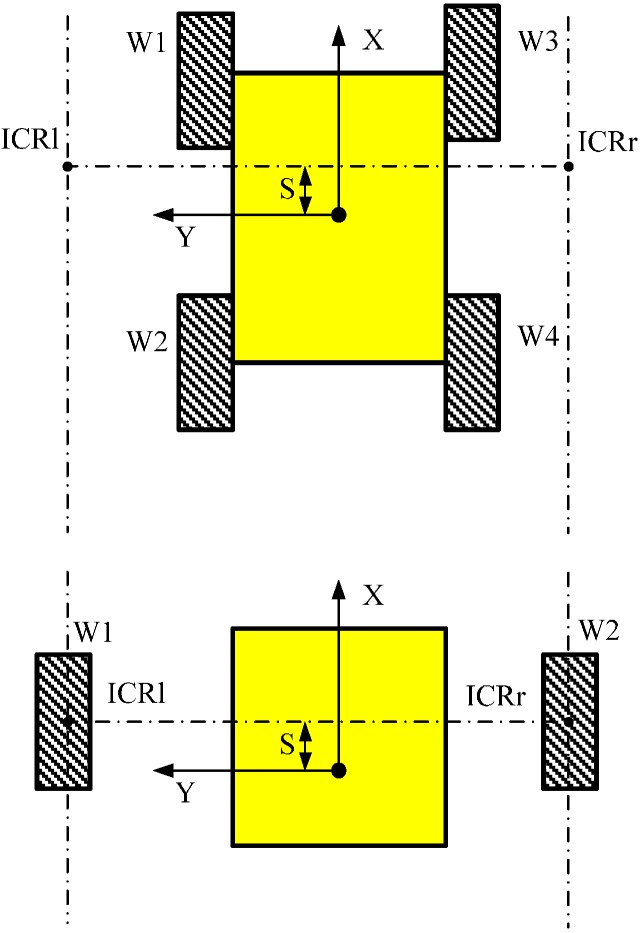
Geometric equivalence between the wheeled skid-steering robot and the ideal differential drive robot.

It is noted that the above expressions also present the kinematics for ideal wheeled differential drive vehicles, as illustrated in Figure 2. Therefore, for instantaneous motion, kinematic equivalences can be considered between skid-steering and ideal wheel vehicles. The difference between both traction schemes is that whereas the ICR values for single ideal wheels are constant and coincident with the ground contact points, tread ICR values are dynamics-dependent and always lie outside of the tread centerlines because of slippage, so we can know that less slippage results in that tread ICRs are closer to the vehicle.

The major consequence of the study above is that the effect of vehicle dynamics is introduced in the kinematics model. Although the model does not consider the direct forces, it provides an accurate model of the underlying dynamics using lump parameters: $ICR_{l}$ and $ICR_{r}$. Furthermore, from assumptions (1) and (3), we get a symmetrical kinematics model, and an ICR coefficient χ from Equation (15) is defined to describe the model. The relationship between ICR coefficient and the vehicle motion path and velocity will be studied.

### 2.2. Dynamic Model for Kinematics Parameters Relationship

#### 2.2.1. Skid-Steering Mobile Robot Dynamic Model

In Section 2.1, the effect of vehicle dynamics is introduced in the kinematics model. This section develops dynamic models of a skid-steering wheeled vehicle for the cases of 2D motion. Using the dynamic models, the relationship between the ICR coefficients and the path and velocity of the vehicle motion will be studied in a simulation.

In contrast to dynamic models described in terms of the velocity vector of the vehicle [4], the dynamic models here are described in terms of the angular velocity vector of the wheels. This is because the wheel velocities are actually commanded by the control system, so this model form is particularly beneficial for motion simulation.

**Figure 3:**
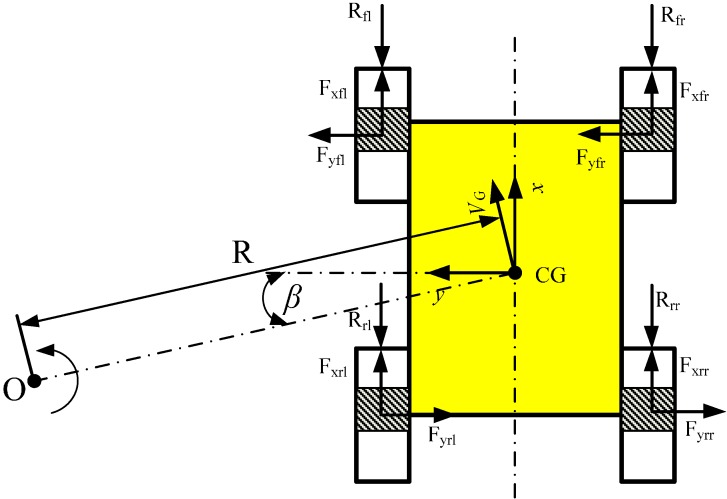
Forces and moments acting on a wheeled skid-steering vehicle during a steady state turn.

As in Figure 3, following Wong’s model [16], the dynamic model is given by:

(16) $$\{\begin{array}{l} F_{xfr}+F_{xrr}+F_{xfl}+F_{xrl}-R_{x}-m\frac{v_{G}^{2}}{R}sin\beta =0\\ F_{yfr}+F_{yrr}+F_{yfl}+F_{yrl}=m\frac{v_{G}^{2}}{R}cos\beta \\ M_{d}-M_{r}=0 \end{array}$$

where $v_{G}$ is the vehicle velocity, and β is the angle between the vehicle velocity and *x*-axis on the local frame. $F_{xfr},\;F_{xrr},\;F_{xfl},\;F_{xrl}$ are the longitudinal (friction) forces and $F_{yfr},\;F_{yrr},\;F_{yfl},\;F_{yrl}$ are the lateral forces. $R_{fr},\;R_{rr},\;R_{fl},\;R_{rl}$ are external motion resistances on the four wheels. $\;M_{d}$ is the drive moment and $\;M_{r}$ is the resistance moment.

Based on the wheel-ground interaction theory [16,17], the shear stress $\tau_{ss\;}$ and shear displacement *j* relationship can be described as:

(17) $$\;\;\tau_{ss\;}=p\mu \left(1-e^{-\frac{j}{K}}\right)$$

where p is the normal pressure, μ is the coefficient of friction and K is the shear deformation modulus.

**Figure 4:**
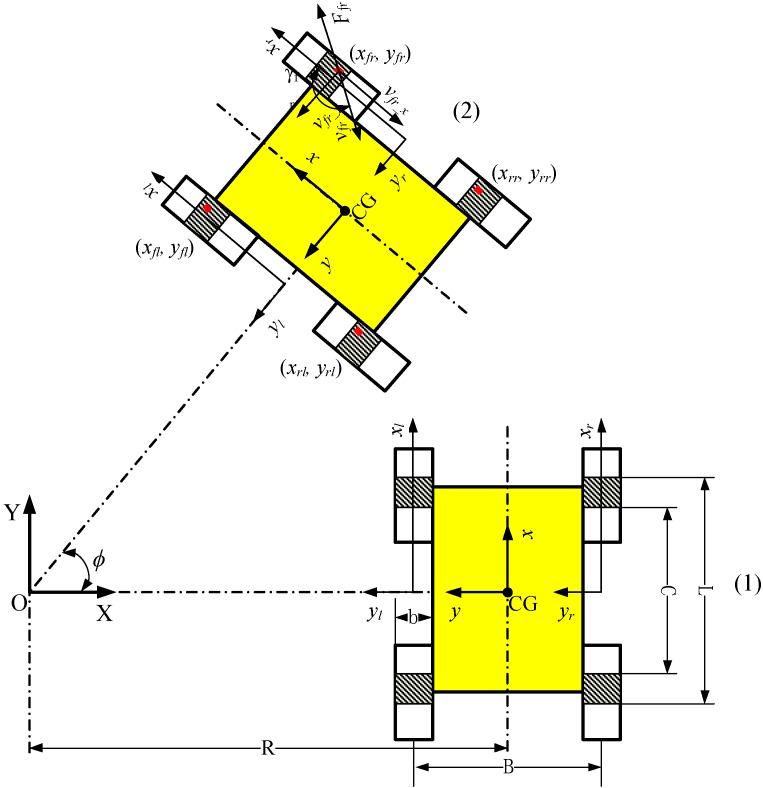
Motion of the skid-steering mobile robot wheel element on the lab surface (firm ground) from position (1) to (2).

Figure 4 depicts a skid-steering wheeled vehicle moving counterclockwise (CCW) at constant linear velocity v and angular velocity $\dot{\phi }$ in a circle centered at O from position (1) to position (2). The four contact patches of the wheels with the ground are shadows in Figure 4. *L* and *C* are the patch-related distances. In the inertial X-Y frame, we define that $j_{fr},\;j_{rr},\;j_{fl},\;j_{rl}$ and $\gamma_{fr},\;\gamma_{rr},\;\gamma_{fl},\;\gamma_{rl}$ are respectively the shear displacements and sliding velocity angle (opposite direction of sliding velocity). The readers can refer to Yu’s work [9] for a detailed analysis. The longitudinal sliding friction and lateral force of the four wheels can be expressed as follows:

(18) $$\{\begin{array}{l} F_{xfr}=\int_{C/2}^{L/2}\int_{-b/2}^{b/2}p_{r}\mu_{r}\left(1-e^{-\frac{j_{fr}}{K_{r}}}\right)sin\left(\pi +\gamma_{fr}\right)dx_{r}dy_{r}\\ F_{yfr}=\int_{C/2}^{L/2}\int_{-b/2}^{b/2}p_{r}\mu_{r}\left(1-e^{-\frac{j_{fr}}{K_{r}}}\right)cos\left(\pi +\gamma_{fr}\right)dx_{r}dy_{r} \end{array},$$

(19) $$\{\begin{array}{l} F_{xrr}=\int_{-L/2}^{-C/2}\int_{-b/2}^{b/2}p_{r}\mu_{r}\left(1-e^{-\frac{j_{rr}}{K_{r}}}\right)sin\left(\pi +\gamma_{rr}\right)dx_{r}dy_{r}\\ F_{yrr}=\int_{-C/2}^{-L/2}\int_{-b/2}^{b/2}p_{r}\mu_{r}\left(1-e^{-\frac{j_{rr}}{K_{r}}}\right)cos\left(\pi +\gamma_{rr}\right)dx_{r}dy_{r} \end{array},$$

(20) $$\{\begin{array}{l} F_{xfl}=\int_{C/2}^{L/2}\int_{-b/2}^{b/2}p_{l}\mu_{l}\left(1-e^{-\frac{j_{fl}}{K_{l}}}\right)sin\left(\pi +\gamma_{fl}\right)dx_{l}dy_{l}\\ F_{yfl}=\int_{C/2}^{L/2}\int_{-b/2}^{b/2}p_{l}\mu_{l}\left(1-e^{-\frac{j_{fl}}{K_{l}}}\right)cos\left(\pi +\gamma_{fl}\right)dx_{l}dy_{l} \end{array},$$

(21) $$\{\begin{array}{l} F_{xrl}=\int_{-L/2}^{-C/2}\int_{-b/2}^{b/2}p_{l}\mu_{l}\left(1-e^{-\frac{j_{rl}}{K_{r}}}\right)sin\left(\pi +\gamma_{rl}\right)dx_{l}dy_{l}\\ F_{yrl}=\int_{-L/2}^{-C/2}\int_{-b/2}^{b/2}p_{l}\mu_{l}\left(1-e^{-\frac{j_{rl}}{K_{r}}}\right)cos\left(\pi +\gamma_{rl}\right)dx_{l}dy_{l} \end{array},$$

where $p_{l}$, $\mu_{l}$ and $K_{l}$ are respectively the normal pressure, coefficient of friction, and shear deformation modulus of the left wheels, and $p_{r}$, $\mu_{r}$ and $K_{r}$ are the ones of the right wheels, respectively. With the other parameters directly measured or given, such as mass of vehicle, m, patch-related distances, *L* and *C*, width of wheel, *b*, $p_{l}$ and $p_{r}$ can be determined by mg/2(L−C)b when a uniform normal pressure distributions assumption used ($p_{l}=p_{r}$). We share some parameters: $\mu_{l},\;\;\mu_{r}$, $K_{l}$ and $K_{r}$ as in Yu’s research [9], because the same platform (Pioneer P3-AT robot) and similar lab surface are used for the simulation.

In Equation (16), the rolling resistance is denoted as $\mu_{roll}$. We can obtain the resistance force, such that:

(22) $$R_{x}=mg\mu_{roll}$$

#### 2.2.2. Dynamic Simulation

This section describes the dynamic simulation that has been used to get the relationship between the treads’ ICRs and path. This model has been simulated for computing the treads’ ICR positions for the Pioneer P3-AT robot. We set all of the key parameters for the model as listed in Table 1.

**Table 1:** The parameters for Pioneer P3-AT robot and terrain dynamic model.

Key Parameters	Symbol	Value
Mass of robot (kg)	*m*	31
Width of robot (m)	*B*	0.40
Length of robot (m)	*L*	0.31
Length of C (m)	*C*	0.24
Radius of tire (m)	*R*	0.11
Width of wheel (m)	*b*	0.05
Shear deformation modulus (m)	$K_{l},K_{r}$	0.00054
Coefficient of rolling resistance	$\mu_{roll,lab}$	0.0371
Coefficient of friction, of $\mu_{sa}$	$\mu_{sa}$	0.4437
Coefficient of friction, of $\mu_{op}$	$\mu_{op}$	0.3093

The values of the ICR coefficient χ and nondimensional path curvature variable λ are computed by solving the non-linear optimization problem with Equations (16)–(22):

(23) $$\underset{\lambda ,\chi }{min}\overset{N}{\underset{i=1}{\sum }}\left[\Delta {F_{xi}}^{2}+\Delta {F_{yi}}^{2}\right]$$

where i denotes the *i*th of N simulated data. When the skid-steering wheeled vehicle is in a constant velocity circular motion, a set of different commanded turning radii is given by:

(24) $$R=\lambda R_{0}\left(m\right),\;\lambda =0,\;1,\;2\;,\ldots ,7$$

**Figure 5:**
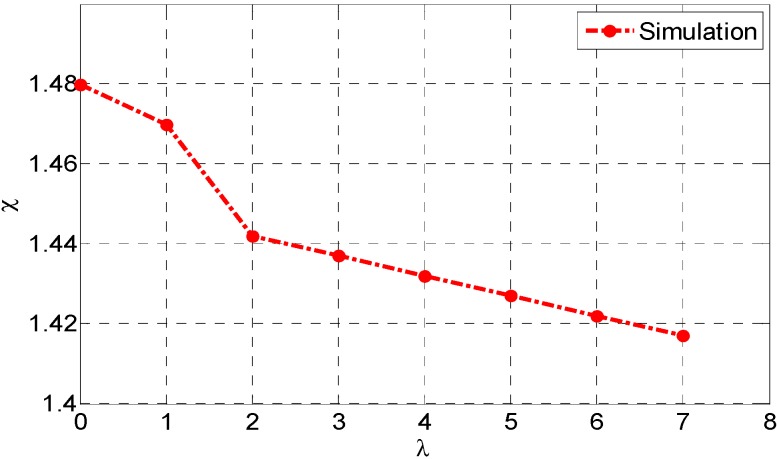
The dynamic simulated results of χ *vs*. λ with Pioneer P3-AT robot using parameters in Table 1.

So we can use Equation (16) to get χ with respect to a special λ. The simulated results of χ *vs.* λ are shown in Figure 5.

In Figure 5, we can find that χ decreases as λ increases, so there is a relationship between these two parameters. In this simulation, we must note that these results are obtained by assuming that the skid-steering mobile robot runs on the firm road and with special values of K, $\mu_{sa}$, and $\mu_{op}$. Because these terrain parameters are difficult to determine, an exact relationship needs further experimental identification.

## 3. Laser-Scanner-Based Experimental Kinematics Method

### 3.1. Proposed Algorithm

In this section, an easy-operating and effective experiment can be used to derive the symmetric kinematics model and the ICR coefficient. When different angular speed control inputs $\omega_{l}$ and $\omega_{r}$ are issued, we consider that the vehicle moves at a constant ICR value, then the following equation can be applied:

(25) $$y_{0}\left(\omega_{l},\omega_{r}\right)=\frac{\int^{\text{​}}\omega_{r}rdt-\int^{\text{​}}\omega_{l}rdt}{2\phi }$$

where ϕ is the actual rotation angle. We can get $y_{0}$ by Equation (25). Then, we can use Equation (15) to determine the ICR coefficient χ.

A set of experiments have been performed. The robot is planned to follow eight different paths with curvatures of radii:

(26) $$R=\lambda R_{0}\left(m\right),\;\;\;\lambda =0,\;1,\;2,\ldots ,\;7$$

Note that λ is denoted in Equation (13). We can choose one of the experimental $y_{0}$ data as $R_{0}$, and on each path, five different speeds:

(27) υ=0.1n(m/s),   n=1, 2, …, 5

With Equations (26) and (27), we can get different $\left(v_{l},v_{r}\right)$ pairs. Therefore, 40 experiments will be performed. The ICR coefficient χ is calculated in each experiment using Equations (15) and (25), which requires measuring the actual speed of each side wheel during the experiment.

### 3.2. Laser-Scanner-Based Localization Method and Experiment Setup

**Figure 6:**
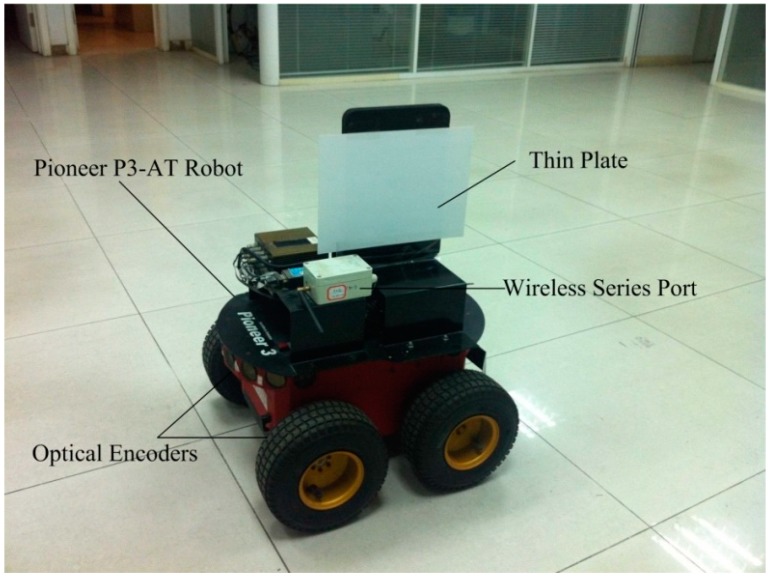
Skid-steering mobile robot platform—a Pioneer P3-AT mobile robot.

The skid-steering mobile robot—a Pioneer P3-AT robot shown in Figure 6—is used for all testing in this research [20], the parameters for the Pioneer P3-AT robot are listed in Table 1 in Section 2.2.2. The P3-AT robot is driven by two motors on each side, and the two wheels of the same side are connected by one chain, so the two wheels of each side rotate at the same speed.

In all of the experiments the field is faced with a tile. Final drive shaft speeds of the motors on the robot are measured using two optical encoders. The optical encoders produce 2048 pulses per resolution, and the interface chip provides quadrature encoding, producing a change of 8192 counts for one revolution, or 0.0439° per count. A YL-100il wireless series port module, which can transmit data transparently, is selected as wireless transmission part. 

**Figure 7:**
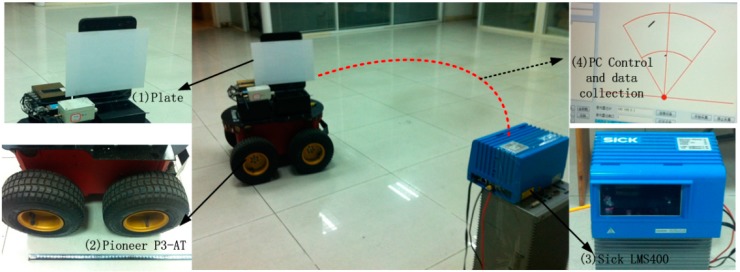
An overview of the proposed localization method based on a Laser Scanner. (**1**) The thin plate; (**2**) Pioneer P3-AT robot; (**3**) Sick LMS400 Laser Scanner; (**4**) Laptop software panel.

A laser scanner-based localization method is used for the position and heading measurements of the robot. An overview of the proposed localization method and the robot control system are demonstrated in Figure 7. In order to localize the robot a thin plate (indicated as (1) in Figure 7) is mounted on top of the Pioneer P3-AT robot in the symmetric plane. A Sick LMS400-10000 laser scanner (3) is installed at the same height, the data of which are used to localize the robot. We use a laptop to control the Pioneer P3-AT robot (4) through a wireless serial port communication. The speed range of the robot is about 0–0.6 m/s.

The Sick LMS400-1000 laser scanner [21], has a large dynamic measurement range of 0.7 m to 3 m with 3 mm systematic error. The field of view of the laser scanner is 70° with 0.1° angular resolution and 270 Hz–500 Hz scanning frequency. The measurement data from the laser scanner are sent to the laptop using an Ethernet connection. The data of the LMS400 regarding the plate are extracted by a clustering method. A line is fitted to the points. The line slope angle is equal to the heading angle, and coordinates of its center are equal to the coordinates of robot geometric center. A median filter, an edge filter and a mean filter are applied to the computed coordinates of robot to smooth the data as much as possible. Measurements of position and heading from the plate can be updated at 135 Hz after filtering. The calculations of position and heading from the plate are shown below. 

**Figure 8:**
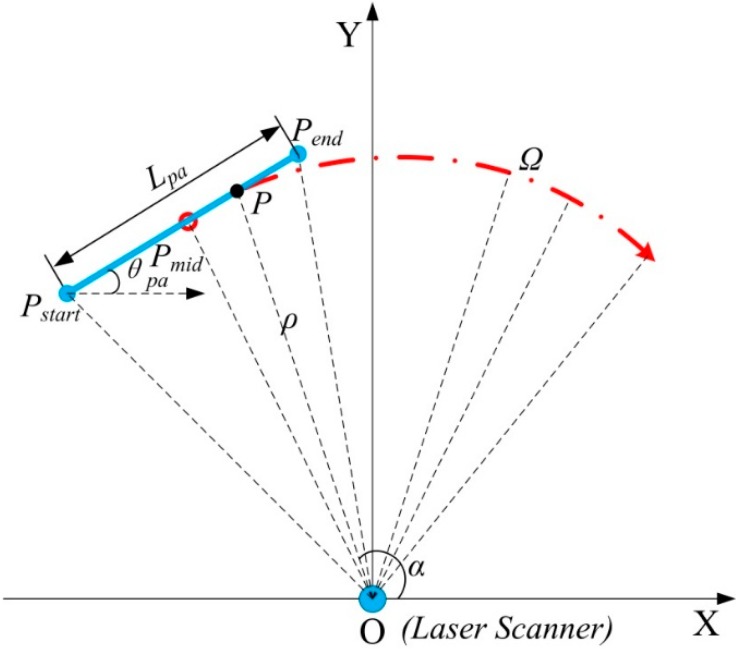
Position and heading measurement from the plate based on LMS400-1000 Laser Scanner for a real trajectory *Ω* of the robot’s center.

In Figure 8, the laser scanner is at origin point O in the inertial frame (X,Y), the actual width of the thin plate is $L_{pa}=290.0\;\text{mm}$, that is from point $P_{Start}$ to $P_{end}$ (the solid blue line) in the laser scanner image region. And $P_{mid}$ is the middle point of plate. The red dot dash line Ω is the planning trajectory of the robot’s center. The coordinates of $P_{mid}$ are the position of the robot’s center coordinates in inertial frame. The angle $\theta_{pa}$ between *X*-axis and line$\;\bar{P_{Start}P_{end}}$, is the heading angle of the mobile robot. We can get point data from the laser scanner from polar coordinates to orthogonal coordinates:

(28) P∙x=ρ cosα, P∙y=ρ sinα

where ρ and α are the distance and corresponding angle of point P measured by the laser scanner, respectively. The measured width of the thin plate $\hat{L_{pa}}$ is:

(29) $$\hat{L_{pa}}=\sqrt{{\left(P_{end}∙x-P_{Start}∙x\right)}^{2}+{\left(P_{end}∙y-P_{Start}∙y\right)}^{2}}$$

All the data on the plate acquired by the laser scanner can be written in orthogonal coordinates:

(30) $$P∙x_{i}=\rho_{i}\;cos\alpha_{i},\;P∙y_{i}=\rho_{i}\;sin\alpha_{i},\;i=1,\;2,\ldots ,n$$

The date can be fitted as a line, and a least-square method is applied to get the heading angle of the line (the same as the robot heading):

(31) P∙y=kP∙x+a

(32) $$P∙\bar{x}=\frac{\sum_{i=1}^{n}P∙x_{i}}{n},\;P∙\bar{y}=\frac{\sum_{i=1}^{n}P∙y_{i}}{n}$$

(33) $$\hat{k}=\frac{\sum_{i=1}^{n}P∙x_{i}∙P∙y_{i}-nP∙\bar{x}∙P∙\bar{y}}{\sum_{i=1}^{n}{\left(P∙x_{i}\right)}^{2}-n{\left(P∙\bar{x}\right)}^{2}}$$

(34) $$\hat{a}=P∙\bar{y}-\hat{k}P∙\bar{x}$$

The line slope angle is equal to the heading angle, considering that the field of view of the laser scanner is 70° (0.389π):

(35) $$\hat{\theta_{pa}}={tan}^{-1}\hat{k},\;\hat{\theta_{pa}}\in \left[-0.194\pi ,\;0.194\pi \right]$$

so we can get the position and heading $q={\left(X,Y,\theta \right)}^{T}$ from the plate based on the laser scanner. The rotation angle ϕ of the mobile robot during interval $t_{end}-t_{0}$ is:

(36) $$\phi =\hat{\theta_{pa}}\left(t_{end}\right)-\hat{\theta_{pa}}\left(t_{0}\right)$$

**Figure 9:**
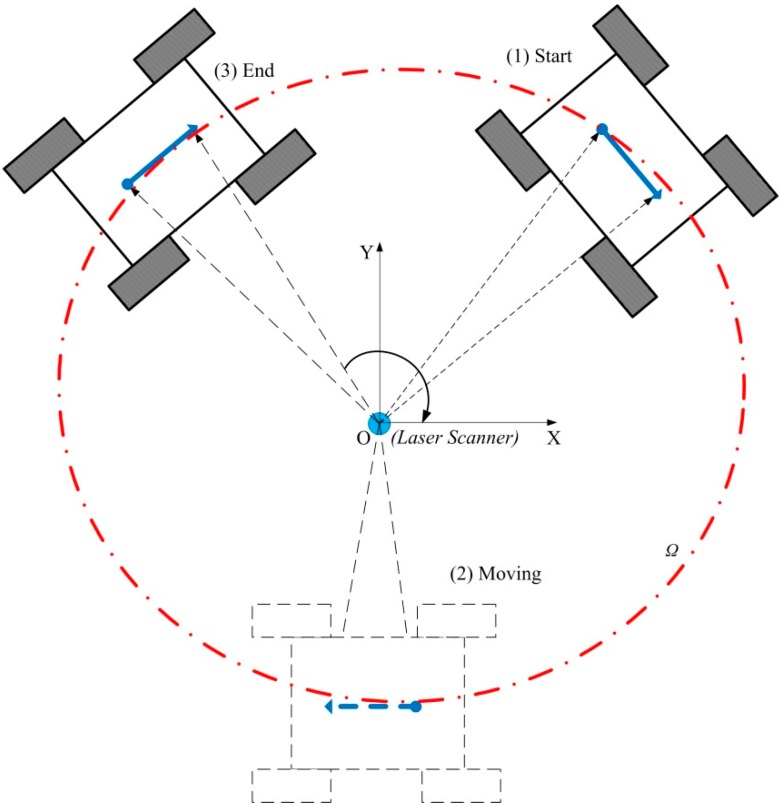
The laser scanner measurement during an entire cycle Ω run from (**1**) start to (**3**) end with the mobile robot.

A set of experiments are executed to get the relationship between the ICR coefficient and the radius of path curvature and speed of the robot. On each path, the skid-steering wheeled vehicle is in constant velocity circular motion. The encoders equipped on the left and right motors give the left and right wheels’ moving distance, that is $\int^{\text{​}}\omega_{l}rdt$ and $\int^{\text{​}}\omega_{r}rdt$. The laser scanner measures the rotation angle change during entire cycle of motion. As in Figure 9, the robot moves clockwise from position (1) to (3), and the laser scanner get the distance data between the thin plate and the laser scanner center at a time interval of 3.7 ms. With Equations (15), (25) and (36), we can get the ICR coefficient χ.

### 3.3. Errors Analysis

The Sick laser scanner measurements errors are given by ∆ρ=0.003 m and $∆\text{α}=\frac{0.1\text{π}}{180}=0.00174\text{ rad}$ [21], so the point data errors in orthogonal coordinates from Equation (28) are:

(37) $$∆P∙x=\left|{\left(\rho \right)}^{\prime }cos\alpha \right|∆\rho +\left|{\left(cos\alpha \right)}^{\prime }\rho \right|∆\alpha \leq ∆\rho +\left|\rho \right|∆\alpha =0.008\;m$$

Similarly, ∆P∙y=0.008 m. When the robot moves, the maximum speed $v_{max}\leq 0.7\text{ m}/\text{s}$, and the laser sample time $∆t_{s}=\frac{1}{f}=\frac{1}{135}s$. The dynamic error when the robot moves is:

(38) $$∆P∙x_{dynamic}\leq v_{max};\;∆t_{s}=0.7\times \frac{1}{135}=0.005\;\text{m}$$

Note that $∆P.x_{static}=∆P∙x$, so the total point data measurement error is:

(39) $$∆P∙x_{total}=∆P∙x_{static}+∆P∙x_{dynamic}=0.013\;\text{m}$$

In Figure 8, with Equations (31), (33) and (36), denote $\phi \in \left[-\frac{\pi }{4},\;\;\frac{\pi }{4}\right]$ and ρ∈[1, 3], so the heading angle ϕ is:

(40) $$\phi =\phi \left(x,y\right)={tan}^{-1}\left(y/x\right),x\in \left[0,\;\frac{3}{\sqrt{2}}],y\in [\frac{1}{\sqrt{2}},\;3\right]$$

and we have the heading angle error:

(41) $$∆\phi =\left|\frac{\partial \phi }{\partial x}\right|∆x+\left|\frac{\partial \phi }{\partial x}\right|∆y=\left|\frac{y}{x^{2}+y^{2}}\right|∆x+\left|\frac{x}{x^{2}+y^{2}}\right|∆y\leq \sqrt{2}∆x=0.018\;rad$$

With Equation (25), we obtain:

(42) $$y_{0}\left(\omega_{l},\omega_{r}\right)=\frac{\int^{\text{​}}\omega_{r}rdt-\int^{\text{​}}\omega_{l}rdt}{2\phi }=f\left(S_{l},S_{r},\phi \right)=\frac{S_{r}-S_{l}}{2\phi }$$

where $S_{r}$ and $S_{l}$ are the displacement distance measured by the left and right wheel encoders, respectively, expressed in millimeters after correction. We note that the position errors are $∆S_{l}$ and $∆S_{\text{r}}$ , $∆S_{l}$ and $∆S_{r}$ are obtained by measuring the actual traveled distance d in straight motion, and the results are $∆S_{l}=0.00026\;\;\text{m},\;∆S_{r}=0.00020\;\;\text{m}$. Note that ∆ϕ=0.018 rad with Equation (41). Let $∆y_{0}$ denote the ICR value error for $y_{0}$:

(43) $$∆y_{0}=\left|\frac{\partial f}{\partial S_{l}}\right|∆S_{l}+\left|\frac{\partial f}{\partial S_{r}}\right|∆S_{r}+\left|\frac{\partial f}{\partial \phi }\right|∆\phi =0.007\;m$$

Note that $y_{0}$ is not less than 0.2 m with a width of robot B=0.40 m. The ICR value error $∆y_{0}=0.007\;\text{m}$, corresponding to extremely small estimate error, is sufficient for further testing.

### 3.4. Results

**Figure 10:**
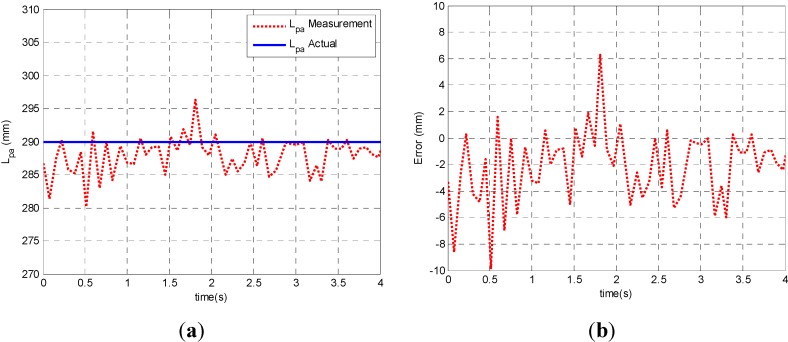
(**a**) A comparison of the measured width of the plate from the laser scanner and the actual value; (**b**) Error between measured width and actual one.

Data are collected in real time and processed off line with MATLAB. Figure 10a shows a comparison of the measured width of the plate from the laser scanner and the actual value. The measured ones, shown as a dot line red line, lies close enough to the actual value (a solid blue line). In Figure 10b, the mean difference between measured width and actual value is shown. In Table 2, it shows that the mean value of measured width μ is 0.289 m and the standard deviation σ is 0.003 m, and the maximum error is −0.01 m, corresponding to an extremely small ICR value estimatation error. The results demonstrate the effectiveness and the feasibility of the proposed laser scanner-based method.

**Table 2:** Mean value and errors of measured width of the plate from the laser scanner.

Test Parameters	μ (m)	σ (m)	Max. Error (m)
Width of the Plate ($L_{pa}$)	0.2890	0.003	−0.01

**Figure 11:**
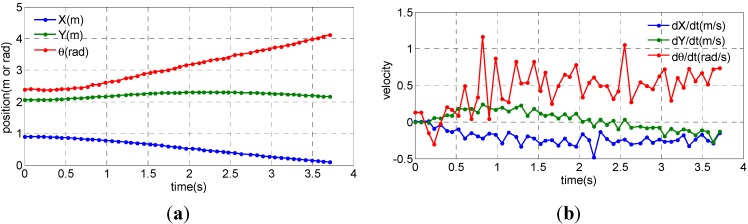
Position and velocity of robots calculated by the proposed algorithm. (**a**) position and heading; (**b**) velocity.

Figure 11 shows the position and heading and velocity of robots calculated by the laser scanner-based localization algorithm during the experiment for speed of 0.25 m/s and curvature radius of 0.475 m.

**Figure 12:**
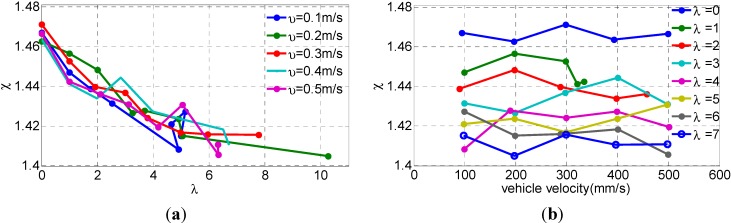
The calculated ICR coefficient *χ* for various: (**a**) path curvatures with v=0.1,  0.2, …0.5 m/s; and (**b**) robot speeds with v=0.1,  0.2, … 0.5 m/s with λ=0, 1, 2, …, 7.

Figure 12 depicts that the ICR coefficient χ for various path curvatures and vehicle velocity. In Table 3, the mean values μ and standard derivations σ of the ICR coefficient χ are shown. It shows that the maximum relative error is less than 1%, and the maximum σ is no larger than 0.01, so the differences between different χ with respect to corresponding λ can be distinguished, with this ICR coefficient measurement accuracy. Furthermore we can find that Figure 12a represents the similar trends as in Figure 5. By increasing the path curvature (increasing λ), the ICR coefficient χ decreases. This implies a specific behavior for χ with respect to λ. Meanwhile, in Figure 12b, the ICR coefficient χ remains almost constant with increasing velocity *v*, at certain λ.

**Table 3:** The calculated ICR coefficient χ for various λ.

Parameters λ	μ	σ	Max. Relative Error
0	1.4662	0.0033	0.34%
1	1.4480	0.0065	0.59%
2	1.4394	0.0055	0.62%
3	1.4341	0.0068	0.71%
4	1.4215	0.0080	−0.92%
5	1.4232	0.0050	0.52%
6	1.4165	0.0077	0.77%
7	1.4115	0.0043	0.30%

In order to reveal the relationship, an approximate function is then used to define χ as a function of the non-dimensional path curvature variable λ:

(44) $$\chi \left(\lambda \right)=1+\frac{a}{1+b{\left|\lambda \right|}^{\frac{1}{2}}}\;,\;\lambda \in \left[0,\;10\right]$$

where *a* and *b* are determined by a curve fit of the experimental data, a>0, b>0. We run 10 sets of experiments with various λ on the lab surface. Numerical values of parameters a=0.4728, b=0.0538 are obtained using a nonlinear least-square algorithm for the function given in Equation (44), and substituted as:

(45) $$\chi \left(\lambda \right)=1+\frac{0.4728}{1+0.0538{\left|\lambda \right|}^{\frac{1}{2}}}\;,\;\lambda \in \left[0,\;10\right]$$

**Figure 13:**
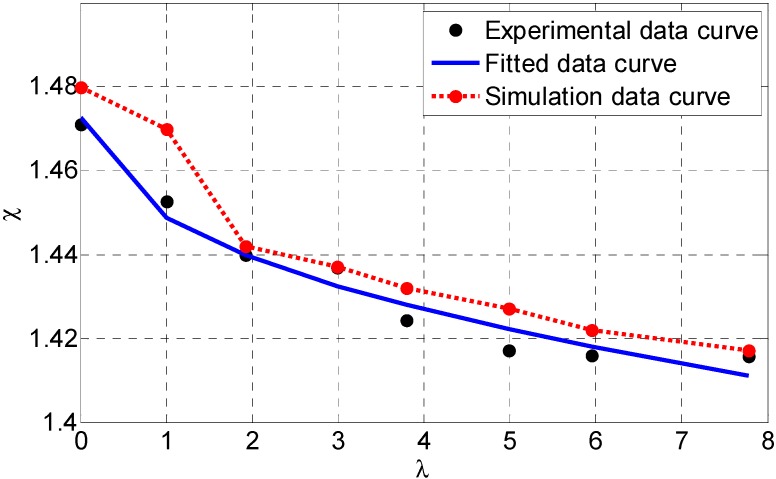
Experimental data curve, simulation data curve and data-fitted curve for χ with respect to λ, when v=0.3 m/s.

Figure 13 shows χ versus λ using the approximating function and experimental data when v=0.3 m/s. The solutions obtained from the simulated and experimental methods are also summarized in this figure. From a qualitative standpoint, experimental results are coherent with those obtained in simulation. The simulation data are a little bigger than experimental results because of uncertain model parameters, e.g., the coefficient of rolling resistance, coefficient of friction, and shear modulus.

### 3.5. Dead-Reckoning Validation

To verify the developed kinematics model with the relationship between ICR coefficient χ and λ, an example path is estimated by two alternative kinematics models: the default P3-AT symmetric model with that χ is a constant value of 1.5, and the proposed approximating model with that χ changes with the nondimensional path curvature variable λ. The example path is different from the ones used in the former model experimental procedure, but it runs in the same environment. 

During the dead-reckoning validation, with Equation (11), we have the default P3-AT kinematics model:

(46) $$\{\begin{array}{l} v_{x}=\frac{v_{l}+v_{r}}{2}\\ v_{y}=0\\ \omega_{z}=\frac{-v_{l}+v_{r}}{2y_{0}}=\frac{-v_{l}+v_{r}}{2B\chi } \end{array}\;\;\;,\chi \equiv 1.5$$

and with Equations (11) and (13), the proposed kinematics model is:

(47) $$\{\begin{array}{l} v_{x}=\frac{v_{l}+v_{r}}{2}\\ v_{y}=0\\ \omega_{z}=\frac{-v_{l}+v_{r}}{2y_{0}}=\frac{-v_{l}+v_{r}}{2B\chi } \end{array}\;,\chi \left(\lambda \right)=1+\frac{0.4728}{1+0.0538{\left|\lambda \right|}^{\frac{1}{2}}}$$

Consider that the center of the robot moves as the following velocity inputs during 6 s. The acceleration is below 3 $\text{m}/{\text{s}}^{2}$, which is nearly the maximum acceleration for the P3-AT mobile robot and the velocity is less than 0.5 m/s, which is nearly the effective maximum velocity of the robot. 

(48) $$\left\{ \begin{array}{l} v_{l}=0,\;\;v_{r}=0,\;\;t=0;\\ v_{l}=0.2,\;\;v_{r}=0.15,\;\;0<t\leq 1;\\ v_{l}=0.2,\;v_{r}=0.1,\;\;1<t\leq 2;\\ v_{l}=0.3,\;v_{r}=0.1,\;\;2<t\leq 3;\\ v_{l}=0.4,\;v_{r}=0,\;\;3<t\leq 4;\\ v_{l}=0.3,\;v_{r}=0.2,\;\;4<t\leq 5;\\ v_{l}=0.3,\;v_{r}=0.25,\;\;5<t\leq 6;\\ v_{l}=0,\;v_{r}=0,\;\;t>6; \end{array}\right.$$

**Figure 14:**
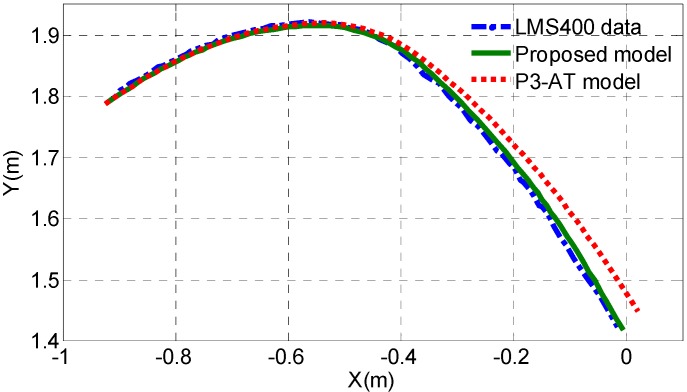
Dead-reckoning performance using proposed model and P3-AT model for an example path with the mobile robot.

Figure 14 shows the estimation of the path based on dead-reckoning (only drive shaft encoders are used) according to the default P3-AT model and the proposed model, and the LMS400 laser scanner data provide the actual position and heading. It can be seen that the proposal model achieves better dead reckoning estimation accuracy than that of the default P3-AT model.

**Figure 15:**
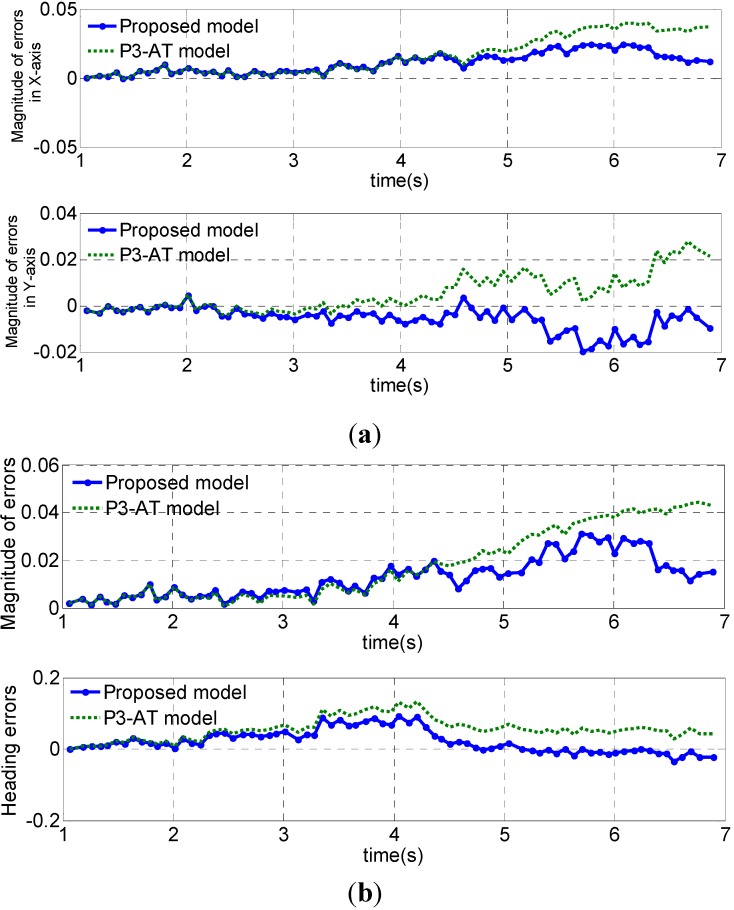
Dead-reckoning errors using the proposed model and the P3-AT model for an example path with the mobile robot. (**a**) Magnitude of position errors in *X* axis and *Y* axis; (**b**) position magnitude errors and heading errors.

Cartesian error and its norm are depicted in Figure 15. The proposed model has a smaller position error within 0.03 m, and a smaller angle error within 0.1 rad. Also, mean squared performance values obtained in the validation path are show in Table 4.

**Table 4:** Mean squared performance values of position and heading errors.

NO.	∆x (m)	∆y (m)	∆Φ (rad)
P3-AT model	0.0169	0.0101	0.0438
Proposed model	0.0273	0.0119	0.0778

**Figure 16:**
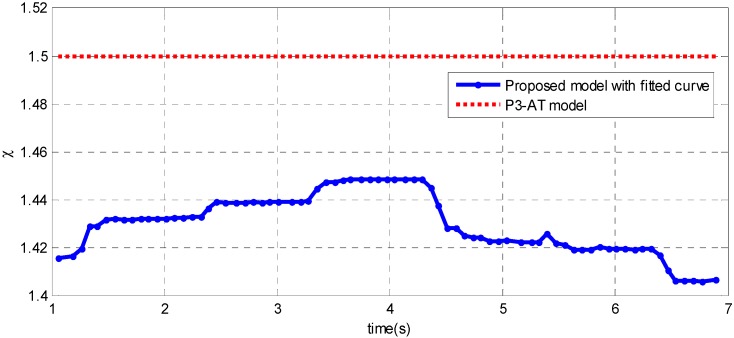
ICR coefficient χ using the proposed model and the P3-AT model for an example path with the mobile robot.

Meanwhile, estimated ICR coefficient χ during the example path is presented in Figure 16. In fact, the ICR coefficient χ varies as the path changes (it is not a constant value of 1.5).

## 4. Conclusions/Outlook

We develop an analysis and experimental kinematics scheme of a skid-steering wheeled vehicle based-on a laser scanner sensor. The ICR coefficient χ and a nondimensional path curvature variable λ are introduced to describe the potential model relationship. The dynamic models based simulation results show that there is some relationship between these two parameters. The laser-scanner-based method experimentally derives the approximating function between χ and λ. The obtained function is validated on a sample path. It was shown that the proposed kinematics model estimated for a skid-steering mobile robot improves the system performance in terms of reduced dead-reckoning errors, with a smaller position error within 0.03 m, and a smaller angle error within 0.1 rad with respect to the default P3-AT model. This test method is easy to operate without adding extra sensors or changing the vehicle mechanical structure and control system. The proposed model and analysis approach can be further used for odometry or to map desired vehicle motion, such as vehicle speed and angular rate, to required wheel speeds.

However, we use the assumptions 2 and 3, which the robot is running on a firm ground surface, and four wheels with the same speed are always in contact with the ground surface. Some mobile robots run on loose soil and in many 4/6 wheels drive (4/6WD) mobile robots, the wheels of each side rotate at different speeds, raising the question of whether a similar result be obtained in a different environment using this method? For future work, it would be interesting to validate the model on different terrains. The current experimental testing results are obtained when acceleration $\text{a}<3\text{m}/{\text{s}}^{2}$ and velocity v<0.5m/s due to the limitations of the robotic platform. Thus, further experiments will be implemented to discuss this problem at higher acceleration and velocity in the future.

## References

[1] Yi J., Wang H.P., Zhang J., Song D. (2009). Kinematic modeling and analysis of skid-steered mobile robots with applications to low-cost inertial-measurement-unit-based motion estimation. IEEE Trans. Robot..

[2] Mandow A., Martinez J.L., Morales J., Blanco J., García-Cerezo A.J., Gonzalez J. Experimental kinematics for wheeled skid-steer mobile robots. Proceedings of IEEE/RSJ International Conference on Intelligent Robots and Systems.

[3] Yi J., Zhang J., Song D., Jayasuriya S. IMU-based localization and slip estimation for skid-steered mobile robots. Proceedings of IEEE/RSJ International Conference on Intelligent Robots and Systems.

[4] Caracciolo L., Luca A.D., Iannitti S. Trajectory tracking control of a four-wheel differentially driven mobile robot. Proceedings of IEEE/RSJ International Conference on Robotics and Automation.

[5] Yi J., Song D., Zhang J., Goodwin Z. Adaptive trajectory tracking control of skid-steered mobile robots. Proceedings of IEEE/RSJ International Conference on Robotics and Automation.

[6] Kozlowski K., Pazderski D. (2004). Modeling and control of a 4-wheel skid-steering mobile robot. Int. J. Appl. Math. Comput. Sci..

[7] Martinez J.L., Mandow A., Morales J., Pedraza S., García-Cerezo A.J. (2005). Approximating kinematics for tracked mobile robots. Int. J. Robot. Res..

[8] Yu W., Chuy O., Collins E.G., Hollis P. Dynamic Modeling of a Skid-Steered Wheeled Vehicle with Experimental Verification. Proceedings of IEEE/RSJ International Conference on Intelligent Robots and Systems.

[9] Yu W., Chuy O., Collins E.G., Hollis P. (2010). Analysis and experimental verification for dynamic modeling of a skid-steered wheeled vehicle. IEEE Trans. Robot..

[10] Maalouf E., Saad M., Saliah H. (2006). A higher level path tracking controller for a four-wheel differentially steered mobile robot. Robot. Auton. Syst..

[11] Kozlowski K., Pazderski D. Practical stabilization of a skid-steering mobile robot—A kinematic-based approach. Proceedings of IEEE 3rd Conference on Mechatronics.

[12] Le A., Rye D., Durrant-Whyte H. Estimation of track-soil interactions for autonomous tracked vehicles. Proceedings of IEEE/RSJ International Conference on Robotics and Automation.

[13] Moosavian S.A.A., Kalantari A. Experimental slip estimation for exact kinematics modeling and control of a tracked Mobile Robot. Proceedings of IEEE/RSJ International Conference on Intelligent Robots and Systems.

[14] Anousaki G., Kyriakopoulos K. A dead-reckoning scheme for skid-steered vehicles in outdoor environments. Proceedings of IEEE/RSJ International Conference on Robotics and Automation.

[15] Anousaki G., Kyriakopoulos K. (2007). Simultaneous localization and map building of skid-steered robots. IEEE Robot. Autom. Mag..

[16] Wong J., Chiang C. A general theory for skid steering of tracked vehicles on firm ground. Proceedings of the Institution of Mechanical Engineers.

[17] Wong J., Wong J. (2001). Theory of Ground Vehicles.

[18] Martinez J.L., Gonzalez J., Morales J., Mandow A., García-Cerezo A.J. (2006). Mobile robot motion estimation by 2D scan matching with genetic and iterative closest point algorithms. J. Field Robot..

[19] Duan Z., Cai Z., Min H. (2014). Robust Dead Reckoning System for Mobile Robots Based on Particle Filter and Raw Range Scan. Sensors.

[20] Technical Description: Pioneer P3-AT Mobile Robot. http://www.mobilerobots.com.

[21] Technical Description: LMS400 Laser Measurement Sensor. http://www.sick.com.

